# Qualitatively different scenarios for co-activation of NMDA, AMPA and GABA receptor currents on dopaminergic neuron

**DOI:** 10.1186/1471-2202-16-S1-P138

**Published:** 2015-12-18

**Authors:** Denis Zakharov, Alexey Kuznetsov

**Affiliations:** 1 Department of Nonlinear Dynamics, Institute of Applied Physics, RAS, Nizhny Novgorod, 603950, Russia; 2 Laboratory of Nonlinear Physics, Nizhny Novgorod State University, Nizhny Novgorod, 603022, Russia; 3 Department of Mathematical Sciences and Center for Mathematical Modeling and Computational Sciences, IUPUI, Indianapolis, IN 46202, USA

## 

We introduced a reduced functional model that qualitatively describes fundamental properties of the dopaminergic (DA) neuron. The neuron is a low-frequency pacemaker, and requires special conditions to increase the frequency of its firing [1]. In more detail, only N-methyl-D-aspartate receptor (NMDAR) current can significantly elevate the frequency (more than 5 fold in comparison with pacemaking). α-Amino-3-hydroxy-5-methyl-4-isoxazolepropionic acid receptor (AMPAR) current is not able to evoke such a high frequency. Instead, it suppresses firing at a conductance much lower than NMDAR. γ-Aminobutyric acid receptor (GABAR) activation first decreases the firing frequency, and only then suppresses firing. Thus, the latter property determines type 1 excitability for the DA neuron, whereas the difference in responses to AMPA and NMDA receptor activation make the neuron different from other well-studied types. We focus this report on the co-activation of these receptors. Co-activation of AMPA together with NMDA receptor may obscure the high frequency firing and lead directly to its suppression. There are contradictory experimental data in this case [2,3]. We show that AMPAR can do both: further contribute to the frequency increase produced by NMDAR, or impede the high-frequency firing. We show that the distinction between these two cases is the level of the background NMDAR activation. Co-activation of GABA together with NMDA receptor may also lead directly to the suppression of high-frequency oscillations (e.g. by shunting inhibition), or reduce the frequency first. Experiments clearly show the latter [4]: the frequency can be reduced back to that in control conditions when GABA and NMDA receptor currents are balanced. This property allows us to recalibrate the model so that it retains type 1 excitability not only in control, but also with activated synaptic currents. In general, activation of synaptic currents changes neural excitability, and we investigate the dynamical mechanisms responsible for the transition. As a result, we were able to construct a model of the DA neuron that collects all the properties described above. The model is essential for further research of behaviorally-relevant modulations of the DA neuron activity, which involves simultaneous excitatory and inhibitory inputs to the neuron.

**Figure 1:**
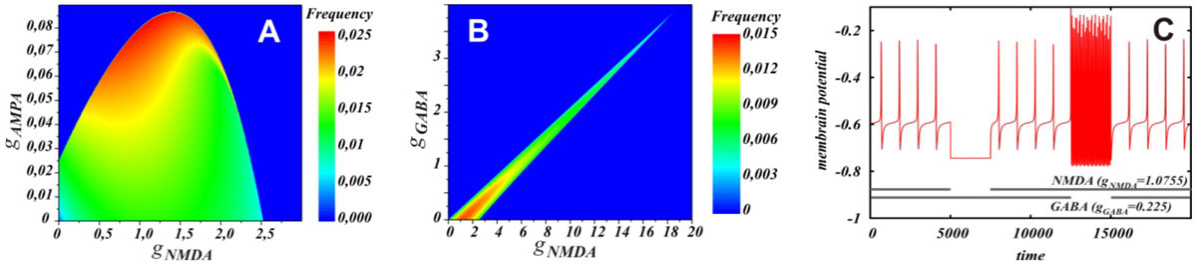
**Influence of the synaptic currents on the neuron activity**. **A **and **B: **dependences of the frequency on the AMPAR and NMDAR, GABAR and NMDAR conductances respectively. C: a voltage trace corresponding to the case of balanced GABAR and NMDAR currents.
